# Insight into the Role of Rb Doping for Highly Efficient Kesterite Cu_2_ZnSn(S,Se)_4_ Solar Cells

**DOI:** 10.3390/molecules29153670

**Published:** 2024-08-02

**Authors:** Chang Miao, Yingrui Sui, Yue Cui, Zhanwu Wang, Lili Yang, Fengyou Wang, Xiaoyan Liu, Bin Yao

**Affiliations:** 1 Key Laboratory of Functional Materials Physics and Chemistry of the Ministry of Education, Jilin Normal University, Siping 136000, China; 2 State Key Laboratory of Superhard Materials, College of Physics, Jilin University, Changchun 130012, China; 3 Key Laboratory of Physics and Technology for Advanced Batteries (Ministry of Education), College of Physics, Jilin University, Changchun 130012, China

**Keywords:** CZTSSe films, Rb doping, sol–gel, solar cells, photoelectric properties

## Abstract

Various copper-related defects in the absorption layer have been a key factor impeding the enhancement of the efficiency of Cu_2_ZnSn(S,Se)_4_ (CZTSSe) solar cells. Alkali metal doping is considered to be a good strategy to ameliorate this problem. In this article, Rb-doped CZTSSe (RCZTSSe) thin films were synthesized using the sol–gel technique. The results show that the Rb atom could successfully enter into the CZTSSe lattice and replace the Cu atom. According to SEM results, a moderate amount of Rb doping aided in enhancing the growth of grains in CZTSSe thin films. It was proven that the RCZTSSe thin film had the densest surface morphology and the fewest holes when the doping content of Rb was 2%. In addition, Rb doping successfully inhibited the formation of Cu_Zn_ defects and correlative defect clusters and promoted the electrical properties of RCZTSSe thin films. Finally, a remarkable power conversion efficiency of 7.32% was attained by the champion RCZTSSe device with a Rb content of 2%. Compared with that of un-doped CZTSSe, the efficiency improved by over 30%. This study offers new insights into the influence of alkali metal doping on suppressing copper-related defects and also presents a viable approach for improving the efficiency of CZTSSe devices.

## 1. Introduction

To achieve a cleaner carbon-neutral future and promote the production of sustainable green energy, abundant, environmentally friendly and non-toxic CZTSSe thin-film solar cells have emerged and are highly competitive among next-generation photovoltaic (PV) technology [1,2,3,4,5]. In addition, kesterite CZTSSe compounds have also been receiving more and more attention because of a higher absorption coefficient (~10^4^ cm^−1^) and adjustable bandgap energy. However, compared to the Shockley–Queisser limit theoretical efficiency (32.2%) [6], current CZTSSe photovoltaic cells only achieve a certified efficiency of 14.9% [7]. Compared to that of other photovoltaic devices, CZTSSe solar cells have a lower open-circuit voltage (V_oc_) [8,9,10], limiting their further widespread application. The main reason for the formation of low open-circuit voltage is the band tail states emerging from a significant amount of Cu_Zn_ defects and [2Cu_Zn_ + Sn_Zn_] defect clusters [11,12,13,14,15].

Investigations have revealed that cationic doping and heterojunction annealing can optimize device performance [16,17,18]. In recent years, cationic doping of alkali metal elements has been validated as a successful strategy for enhancing the PCE of CZTSSe devices [19,20,21]. Studies have also shown that Li, Na, and K elements can be successfully doped into the CZTSSe crystal structure and thus reduce V_oc_ loss and improve cell efficiency. Rubidium, as the same main group element, is known as an effective dopant in enhancing the performance of Cu(In,Ga)Se_2_ (CIGS) thin-film photovoltaic devices [22,23]. However, in the domain of CZTSSe, rubidium-doped CZTSSe have only been prepared by magnetron sputtering. Wu et al. prepared CZTSSe thin films with different Rb content by magnetron sputtering, and proved that Rb doping can reduce the formation of the high-resistance ZnSe phase and improve device efficiency [24]. Compared with magnetron sputtering, the sol–gel method is one of the simple, economically, and efficient methods, and has its own advantages like low processing temperature, non-vacuum processing conditions, and homogeneity of the produced material. However, there have been no studies conducted on the synthesis of the RCZTSSe absorption layer material using the sol–gel technique, and the effect of Rb doping on the efficiency of CZTSSe devices so far.

In this study, we successfully synthesized high-quality RCZTSSe thin films through a straightforward sol–gel technique. The characteristics of the RCZTSSe thin films, including their structure, optical properties, and electrical behavior, were thoroughly examined. The results show that the applicable content of Rb doping could enhance the quality of the crystallization of the absorption layer and carrier mobility. Moreover, the doping of a small amount of Rb could further suppress the formation of Cu_Zn_ defects and correlative defect clusters. When the content of Rb was 2%, device efficiency reached 7.32% and open circuit voltage increased to 379.69 mV.

## 2. Results and Discussion

### 2.1. Structural Characterization

**Figure 1:**
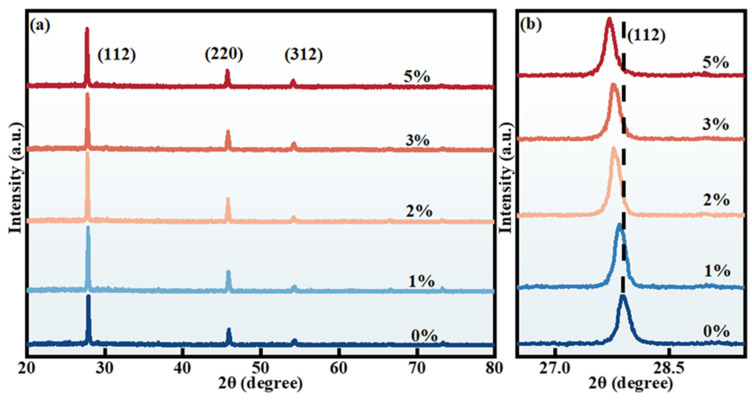
(**a**) XRD spectra of RCZTSSe thin films with various Rb contents of 0%, 1%, 2%, 3%, and 5%. (**b**) Enlarged image of the (112) diffraction peaks.

Firstly, XRD data of RCZTSSe thin films with diverse Rb-doping content were analyzed. The results are shown in Figure 1a. In the following, for convenience of description, Rb-0%, Rb-1%, Rb-2%, Rb-3%, and Rb-5% were used to represent the un-doped sample and the doped samples with 1%, 2%, 3%, and 5% of Rb-doping content, respectively. XRD diffraction peaks of all films were located at 27.20°, 45.26°, and 53.39°, corresponding to (112), (220), and (312) crystallographic planes of CZTSSe, respectively [25]. Besides the three peaks above, other diffraction peaks of copper-, zinc-, selenium-, and rubidium-related complexes were not observed, indicating that Rb atoms entered the crystal lattice in the form of replacing Cu atoms or gap atoms, and no impurity phase was formed on the film surface. Figure 1b shows the corresponding enlarged image of the (112) diffraction peaks. When the Rb-doping content increased from 0% to 5%, the diffraction peak moved towards a smaller angle. The reason was that the Rb^+^ ion’s radius (1.52 Å) surpassed that of the Cu^+^ ion (0.77 Å), and the replacement of Cu^+^ by Rb^+^ ion was the cause of the expansion of the volume of a single crystal cell.

**Figure 2:**
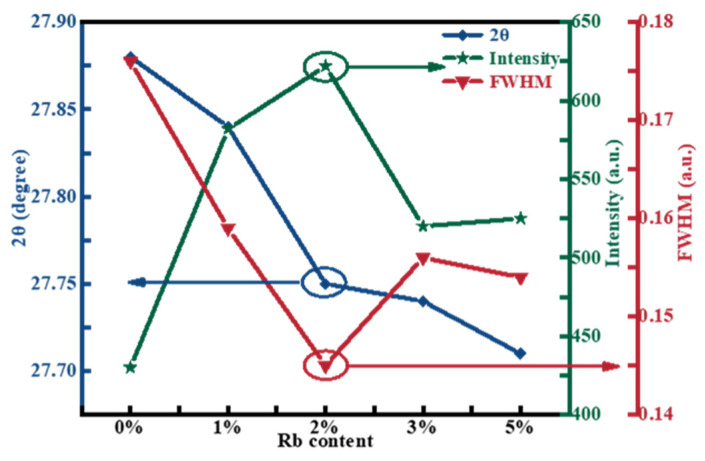
Variation trend in 2θ, FWHM, and intensity of (112) peaks with various Rb-doping contents of RCZTSSe films.

To further research the effect of Rb-doping content on the crystal quality of CZTSSe thin films, the full width at half maximum (FWHM), the diffraction angle 2θ, and the intensity of the peak were obtained from the (112) peak in the XRD spectrum, as shown in Figure 2. The slight shift of the (112) diffraction peak toward a smaller angle was observed with the Rb content increasing. It was because the replacement of smaller Cu^+^ ions by larger Rb^+^ ions [26]. When the Rb-doping content was in the range of 0% to 2%, the (112) peak intensity of the film displayed an increasing tendency, and FWHM fell from 0.176 to 0.145, indicating that the crystallinity increased with increasing Rb content. However, as the doping content continue to rise, the intensity of the (112) peak reduced, and FWHM increased gradually, indicating that the crystallinity of the absorbed layer decreased. It was concluded that the crystallinity of the film samples was optimal when the Rb-doping content was 2%.

According to the XRD results, the crystal lattice parameters were calculated by using the Bragg equation, and the influence of Rb doping on the crystal structure was discussed. The Bragg equation for tetragonal crystal materials such as CZTSSe can be expressed in terms of the Miller index (hkl) [27]:

(1) $$\sin\theta^{2}=\frac{\lambda^{2}}{4a^{2}}(h^{2}\;+\;k^{2})\;+\;l^{2}$$

The Bragg equation was utilized in conjunction with Equation (1) to obtain Equation (2), and the ultimate lattice constant is denoted by Equation (3):

2d_hkl_sinθ = nλ (2)

(3) $$\frac{1}{{d^{2}}_{\mathrm{hkl}}}=\frac{h^{2}+k^{2}}{a^{2}}+\frac{1^{2}}{c^{2}}$$

**Figure 3:**
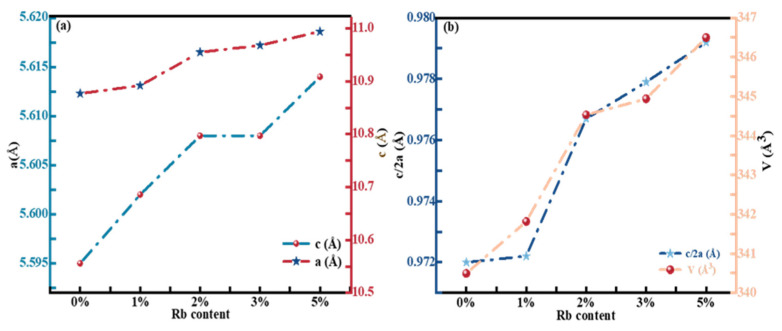
(**a**) Lattice constants a and c of the RCZTSSe thin films with different Rb contents of 0%, 1%, 2%, 3%, and 5%. (**b**) ƞ and V of the RCZTSSe thin films with various Rb-doping contents.

In the formula, d_hkl_ represents lattice spacing (hkl). The lattice constant a can be ascertained from the (220) plane, while the lattice constant c is derivable from the (112) plane. Figure 3a displays the lattice constants of the RCZTSSe films with various Rb contents of 0%, 1%, 2%, 3%, and 5%. It can be seen that when Rb-doping content increased, a escalated from 5.61 Å to 5.62 Å and c increased from 10.56 Å to 10.91 Å. It was due to Rb^+^ having a bigger ionic radius that primarily occupied the site of Cu^+^, which has a smaller ionic radius, in the RCZTSSe thin film, leading to an increase in the lattice constant [28], which was consistent with XRD results. In order to prove the structure of the RCZTSSe film, Figure 3b displays the lattice parameters η and volume V of the RCZTSSe film. According to previous reports, when the lattice parameter η is less than or equal to 1, the corresponding CZTSSe-based film sample exhibits a kesterite structure. Figure 3b demonstrates that the η value of RCZTSSe remained below 1 as Rb content increased from 0% to 5%. It is indicated that the crystal structure of the thin film remained unchanged after Rb doping. Furthermore, the V value increased with increasing Rb-doping content due to the larger ionic radius of Rb^+^ compared to that of Cu^+^, providing further evidence of successful Rb doping into the CZTSSe lattice.

**Figure 4:**
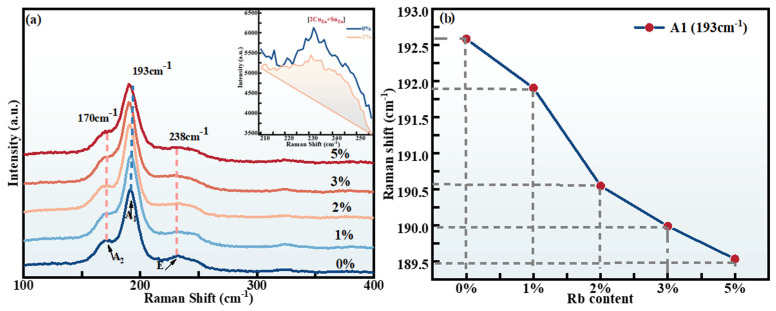
(**a**) Raman spectra of the RCZTSSe thin films with different Rb-doping contents, and enlarged Raman spectra of the Rb-0% and Rb-2% RCZTSSe thin films in the range of 210–255 cm^−1^. (**b**) The variation tendency chart of the primary Raman peaks of the A1 mode with different Rb-doping contents.

However, although no diffraction peak of secondary phases was found in the XRD spectrum, the existence of Cu_2_SnS_3_, Cu_2_SnSe_3_, ZnSe, and ZnS impurity phases still could not be ruled out due to their diffraction peaks coinciding with those of CZTSSe. Therefore, Raman scattering was used as an auxiliary method to detect these possible impurity phases. Shown in Figure 4a, the significant vibrations at 170, 193, and 238 cm^−1^ in the Raman spectra are closely related to CZTS and CZTSe and are characteristic modes (A2, A1, and E) of the kesterite structure, respectively [29]. Therefore, all peaks appearing in the spectra were associated with CZTSSe materials, and other impurity phases were not found.

In addition, a change in Raman peak intensity can reflect the variation in defect clusters in CZTSSe. The variation of peak intensity at 236 cm^−1^ is positively correlated with the defect concentration of the [2Cu_Zn_ + Sn_Zn_] defect clusters [30,31]. The illustration in Figure 4a shows the enlarged Raman spectra of Rb-0% and Rb-2% samples in the range of 210–255 cm^−1^. Comparing with that of the Rb-0% film, the peak intensity of the Rb-2% thin film at 236 cm^−1^ diminished slightly, suggesting a reduction of the adverse [2Cu_Zn_ + Sn_Zn_] defect cluster density. Combined with XRD and Raman analyses, it was found that Rb doped into the lattice of CZTSSe could impede the formation of detrimental deep-level defects by occupying Cu position individually. Figure 4b reveals the change of the peak position of the A1 vibration mode with Rb-doping content, so as to explore the impact of Rb-doping content on the Raman spectrum. As the content of Rb doping increased from 0% to 5%, the primary Raman peak of the RCZTSSe films exhibited a red shift. It was because larger Rb ions were incorporated into the CZTSSe lattice, resulting in lattice expansion, which led to changes in atomic vibration. These findings are consistent with the results from XRD analysis.

**Figure 5:**
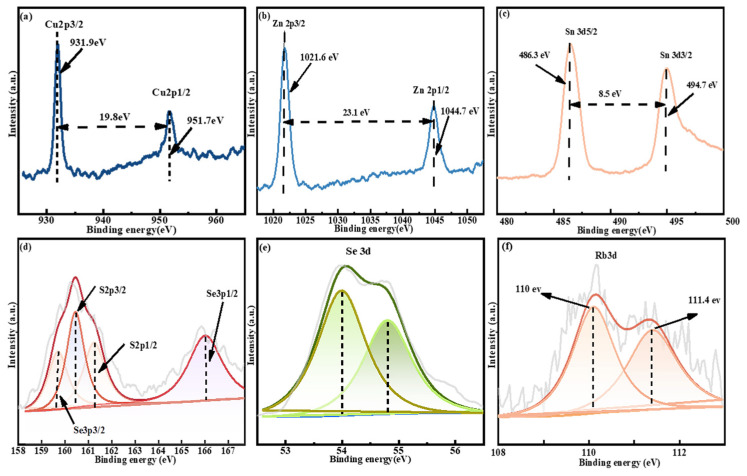
XPS spectra of (**a**) Cu 2*p*, (**b**) Zn 2*p*, (**c**) Sn 3*d*, (**d**) S 2*p*, (**e**) Se 3*d*, and (**f**) Rb 3*d* of the RCZTSSe 0thin film.

Subsequently, to ascertain the valence states of Cu, Zn, Sn, S, Se, and Rb in RCZTSSe, we employed XPS measurements, illustrated in Figure 5. After cation replacement, the valence states of Cu(+1) and Zn(+2) can be determined from the peak positions and energy differences in the Cu 2*p* and Zn 2*p* spectra shown in Figure 5a,b [32,33]. The XPS spectrum of Sn 3*d* is depicted in Figure 5c. Two independent peaks situated at 486.3 eV and 494.7 eV were consistent with the core energy levels of Sn 3*d_5/_*_2_ and Sn 3*d*_3/2_, respectively. This suggests that Sn is in a +4 valence state [34]. Since the fitting curve of S 2*p* nearly overlapped with that of Se 3*p*, the S 2*p* XPS spectrum was acquired using Gaussian fitting techniques, as shown in Figure 5d. These characteristic peaks obtained after fitting were matched with Se 2*p*_3/2_, S 2*p*_3/2_, S 2*p*_1/2_, and Se 3*p*_1/2_. The peak values of S 2*p*_3/2_ and S 2*p*_1/2_ were 160.5 eV and 161.3 eV, respectively, within the established range of standard values for S in sulfide compounds. Consequently, it was deduced that S was present as S^2−^ in RCZTSSe [35]. Figure 5e shows that the peaks of Se 3*d*_3/2_ and Se 3*d*_1/2_, identified using the Gauss-Lorentz fitting technique, were positioned at 54.8 and 54.0 eV, respectively. This indicates that Se ions were present in the form of Se^2−^ in RCZTSSe [36]. Figure 5f also shows the existence of Rb in the film. Rb 3*d* peaks were recorded at 110 eV and 110.4 eV, and its binding energy was nearly equivalent to that of Rb^+^. It indicated that the valence state of Rb was +1 [37]. Therefore, the XPS results showed that Rb was successfully doped into CZTSSe, and the composition elements of RCZTSSe film samples were obtained in the chemical valence states of Cu^+^, Zn^2+^, Sn^4+^, S^2−^, Se^2−^, and Rb^+1^.

**Figure 6:**
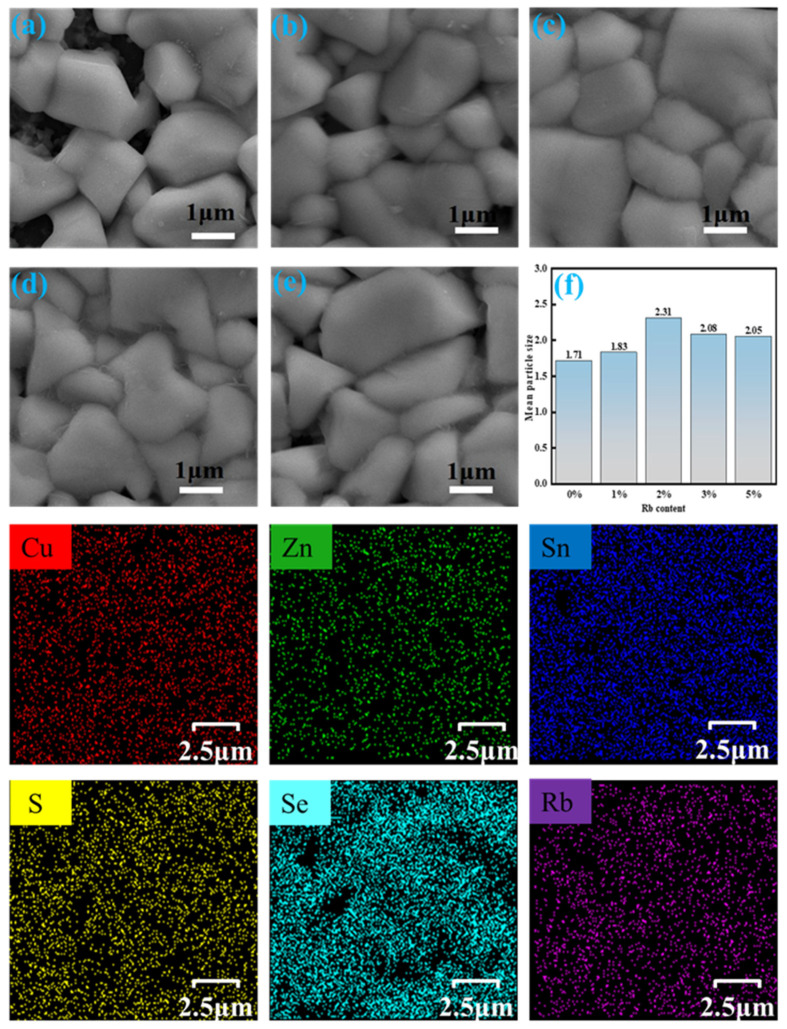
SEM images of RCZTSSe films with various Rb contents: (**a**) Rb-0%, (**b**) Rb-1%, (**c**) Rb-2%, (**d**) Rb-2%, and (**e**) Rb-5%. (**f**) The average grain dimensions in the films. EDS mapping images of Cu, Zn, Sn, S, Se, and Rb elements in the Rb-2% thin film.

A high-quality CZTSSe absorbing layer with high crystallinity and a smooth and dense morphology is also key to achieving high-performance devices [38]. Therefore, we ulteriorly studied the effect of Rb doping on the growth of grains. In Figure 6, the morphology of RCZTSSe films characterized by SEM is shown. It is clearly discernible from Figure 6a–e that the dimension of the grains of all RCZTSSe film samples increased compared with that of the un-doped CZTSSe film sample [39]. The Rb-0% thin film sample had a rough surface, small grain size, and many holes. The existence of holes may hinder the transfer of charge, which harmfully influences the performance of the device. When the doping amount of Rb increased from 0% to 2%, the film sample became inceasingly flatter, denser, and smoother. It is observable in Figure 6f that the average grain dimension of the Rb-2% thin film sample was the largest. It has been reported that in the early selenization process, the elements of alkali metals in the precursor film can potentially react with Se to form alkali metal–Se liquid phase melts. These liquid phase melts can hasten the spread of Cu, Zn, and Sn ions, thereby improving the crystallinity of the absorption layer and passivating the grain boundaries [40,41]. Thus, in the present work, a Rb–Se liquid phase may have been generated after Rb doping, which was conducive to the change of film sample morphology and the inhibition of recombination loss at the grain boundary. However, Figure 6d,e shows that with an incremental rise in Rb content, the surface of the film progressively became coarse, and voids also increased, which was inconvenient to carrier transport [42]. Therefore, suitable Rb doping can enhance grain development, improve the morphology of the film, and aid in the preparation of efficient RCZTSSe solar cells. Finally, we found that when the doping content of Rb was 2%, the surface of the RCZTSSe thin film was exceptionally flat, smooth, and highly compact. Additionally, the elements distribution diagram of the Rb-2% thin film measured by EDS is shown in Figure 6. It can be observed that Cu, Zn, Sn, S, Se, and Rb were evenly distributed in RCZTSSe.

Element composition alteration in the film is very important to crystallinity and the distribution of RCZTSSe defects. Using EDS, the constitution and distribution of elements in the film were analyzed. Table 1 enumerates the actual element composition in RCZTSSe with various Rb-doping contents detected by EDS. It is shown in Table 1 that the Rb/(Rb + Cu) ratios in the films increased with the increasing Rb doping ratio in the solutions. This indicates that the atomic proportion Rb/(Rb + Cu) of RCZTSSe could be modified through altering the chemical proportions of the solutions. Every sample demonstrated Cu-poor and Zn-rich proportions. As is common knowledge, the Cu-poor and Zn-rich ratio can enhance the formation V_Cu_ and restrain the formation of Cu_Zn_, which have the effect of maximizing the PCE of CZTSSe devices [43].

**Figure 7:**
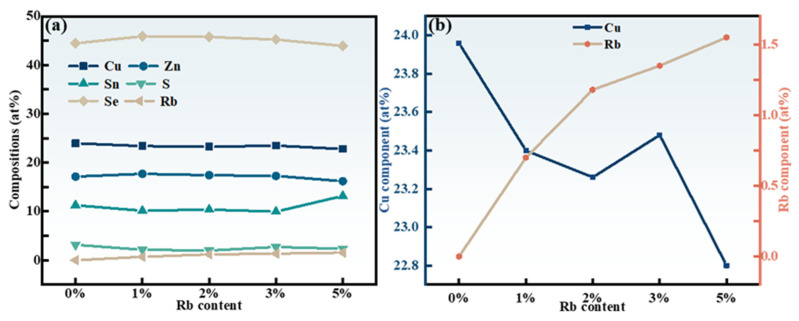
(**a**) Atomic percentage of all elements in RCZTSSe films with different Rb contents. (**b**) Ratio of elemental composition of Rb and Cu in RCZTSSe films with different Rb contents.

Figure 7a displays the EDS outcomes of the RCZTSSe films, also shown in Table 1. The findings indicate that with an increasing Rb doping ratio, the content of other elements except Cu in the film were finally actually unchanged. Figure 7b demonstrates that the proportion of Cu atoms decreased when the content of Rb gradually increased. The above results indicate that Rb doped into the CZTSSe lattice by substituting the partial Cu atom.

### 2.2. Photoelectric Characteristics

**Figure 8:**
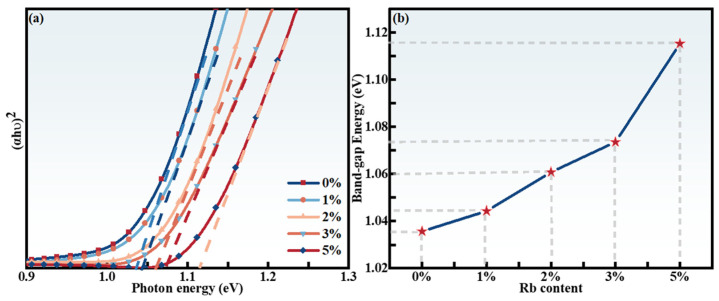
(**a**) (αhυ)^2^ versus photon energy (hυ) for the RCZTSSe thin films with diverse Rb contents (Rb-0%, Rb-1%, Rb-2%, Rb-3%, and Rb-5%). (**b**) Variation trend of bandgaps of the RCZTSSe films with Rb content.

For the purpose of investigating the impact of Rb-doping content on the optical bandgap (E_g_) of RCZTSSe thin films, a UV–Vis–NIR spectrophotometer was utilized to examine the absorption spectra of the RCZTSSe films. Figure 8a shows the (αhυ)^2^-hυ curve of the RCZTSSe films with diverse Rb-doping content. The optical E_g_ value of RCZTSSe was determined using the Tauc equation, based on the solid-state energy band theory [44]:

αhυ = B(hυ − E_g_)^n^ (4)

where α, hυ, and B are the absorption coefficient, photon energy, and Planck’s constant, respectively [45]. Theoretical research suggests that for permitted direct transition, permitted indirect transition, prohibited direct transition, and prohibited indirect transition semiconductors, the corresponding values of n are 1/2, 3/2, 2, and 3, respectively. RCZTSSe films are direct bandgap semiconductors, therefore, n was 1/2. The correlation between α and the bandgap can be expressed with the listed formula:

αhυ = B(hυ − E_g_)^1/2^ (5)

To obtain the value of E_g_, the linear part of the curve (αhυ)^2^ was projected onto the X-axis (i.e., (αhυ)^2^ = 0, Eg = hυ). As shown in Figure 8b, the E_g_ values of RCZTSSe films with various Rb contents (Rb-0%, Rb-1%, Rb-2%, Rb-3%, and Rb-5%) were 1.03, 1.04, 1.06, 1.07, and 1.11 eV, respectively. The RCZTSSe thin films’ bandgaps increased with increasing Rb-doping content. This shows that Rb doping could obtain continuously tunable E_g_. The bandgap value refers to the variance in energy between the valence band maximum (VBM) and the conduction band minimum (CBM) (i.e., CBM = VBM + E_g_) [46]. Based on the first-principles calculations, for RCZTSSe films, the hybridization of Cu 3*d* and anionic S 3*p* (Se 4*p*) orbitals control the VBM. The position of the CBM is ascertained by the hybridization of the Sn *5s* and S 3*p* (Se 4*p*) orbitals. As reported in some articles, the stronger hybridization between the Cu 3*d* and S 3*p* states can push the VBM upward [47,48]. However, this stronger hybridization is weakened when part of Cu^+1^ is replaced by Rb^+1^. In other words, the VBM tends to decline with the doping of Rb. Replacing Cu with Rb results in a decrease in the VBM, and the CBM remains invariable, thus, the bandgap of the absorption layer in RCZTSSe film can be modified through the regulation of Rb-doping content. According to literature reports, an absorbing layer with a large bandgap is expected to provide high V_oc_ and device performance because it can suppress recombination at the heterojunction interface [49,50]. Therefore, we believe that Rb doping has a beneficial impact on improving the performance of CZTSSe films.

To better comprehend the electrical properties of RCZTSSe films at room temperature, Hall measurements were performed for RCZTSSe films with different Rb contents, as shown in Table 2. Since a Cu_Zn_ defect is the primary acceptor defect in CZTSSe films, CZTSSe films show p-type conductivity. With Rb-doping content increasing from 0% to 5%, all RCZTSSe thin film samples still showed p-type conductivity, as evidenced in Table 2. However, the concentration of carriers decreased from 6.26 × 10^17^ cm^−1^ to 1.27 × 10^17^ cm^−1^ and subsequently increased to 7.28 × 10^18^ cm^−1^. As Rb content increased, the concentration of the carriers fells first, then roses, relying on the composition of the film or defects. The primary sources of carriers in CZTSSe are defects. Consequently, when a significant quantity of defects and associated defect clusters are present in the films, it can give rise to a higher carrier concentration [51]. A decline in carrier concentration suggests that Rb doping serves as a restraining factor in the formation of defects. Meanwhile, mobility first decreased and then increased as Rb-doping content increased. Firstly, Hall mobility increased from 2.23 × 10^1^ cm^2^ V^−1^s^−1^ to 6.64 × 10^1^ cm^2^ V^−1^s^−1^ with the increase in Rb-doping content from 0 to 2%. This may have been due to the improved crystallinity of the film, which formed a dense, no-holes, and large-grain morphology, making it easier for carriers to transport [52]. In addition, the reduction of carrier concentration resulted in a weaker collision and scattering process, which boosted carrier mobility. When Rb-doping content was 2%, Hall mobility was the highest. However, when the content of Rb continued to increase, Hall mobility decreased to 7.03 × 10^1^ cm^2^ V^−1^s^−1^. According to SEM, when the content of Rb was too high, it was observed that holes emerged on the surface of films and the crystal quality of the film became worse, which was not beneficial to carrier transport.

### 2.3. Device Characterization

**Figure 9:**
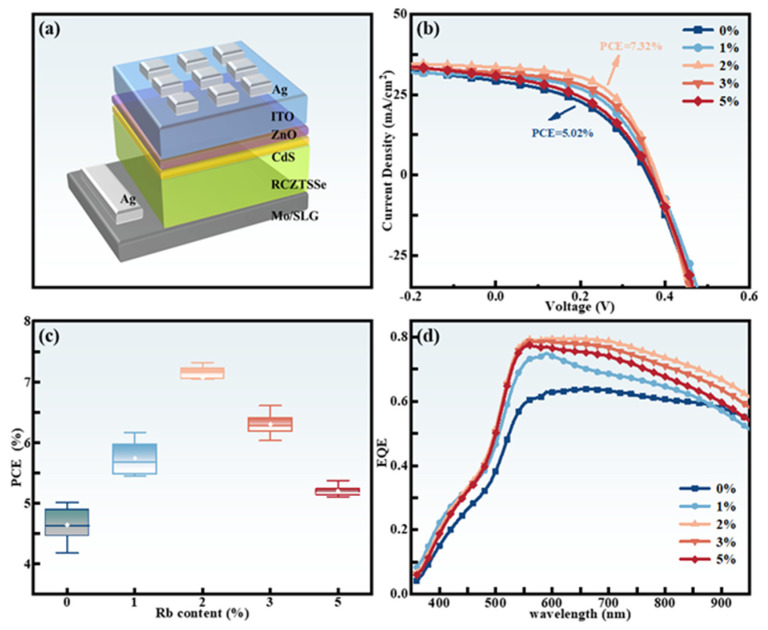
(**a**) The diagrammatic configuration of the RCZTSSe solar cell. (**b**) Optimal J–V curves of RCZTSSe devices with various Rb-doping contents under standard AM 1.5 sunshine. (**c**) Box diagrams of PCE derived from 15 s olar cells. (**d**) EQE spectrum of the RCZTSSe solar cells.

To understand the influence of Rb doping on the performance of CZTSSe solar cells, devices with a Ag/ITO/i-ZnO/CdS/RCZTSSe/Mo/SLG structure were prepared, as shown in Figure 9a. RCZTSSe solar cells with varying Rb-doping content were adopted for analysis in detail under simulated AM 1.5 sunshine. Figure 9b displays the J–V curves of the five devices, from which their photovoltaic and electrical characteristics were derived, as showed in Table 3. The J–V curve shows that the device with 2% Rb content exhibited superior photovoltaic characteristics. The maximum PCE of 7.32% was attained by the champion photovoltaic device. Table 3 indicates that, compared to those of the CZTSSe device, the V_oc_, J_sc_, and fill factor (FF) of all RCZTSSe solar cells showed substantial improvements. On the one hand, the increase in V_oc_ might have stemmed from the reduction of Cu_Zn_ defects and [2Cu_Zn_ + Sn_Zn_] defects from Rb doping, easing the intense band tail states [53]. On the other hand, substituting Cu with Rb might have enabled an adjustment of the valence band edge, causing better alignment of bands between the absorbing and buffer layers, thereby lessening the V_oc_ losses induced by interface mismatch [48,54]. The increase of grain dimensions and crystallinity of the absorbing layer is the reason for the improvement in J_sc_ and FF [55]. According to Table 3, with an increase in the Rb-doping content from 0% to 5%, the series resistance (R_s_) diminished initially, followed by an increase. However, the variation trend of the shunt resistance (R_sh_) was contrary to that of R_s_. The minimum value of R_s_ was 12.66 Ω·cm^2^, and the maximum value of R_sh_ was 690.58 Ω·cm^2^ when the content of Rb was 2%. We know that diminishing the number of holes in the RCTSSe absorption layer and an improvement of film smoothness have a positive impact on the RCTSSe/CdS interface, which can suppress the recombination of photogenerated carriers, thereby lessening R_s_ [56]. Moreover, the increased R_sh_ was due to Cu_Zn_ and [2Cu_Zn_ + Sn_Zn_] defects being suppressed by Rb doping and inhibited shunt effect. Therefore, the changes in R_s_ and R_sh_ contributed to the further optimization of J_sc_ and FF.

To guarantee the precision of the experiment, 15 cells were chosen for statistical analysis at each Rb-doping content. The PCE characteristics of solar cells with variable Rb-doping content were assessed using the box diagram (Figure 9c). It was also evident that the PCE of the device was enhanced after Rb doping. In the case of ideal Rb-doping content (i.e., the content of Rb was 2%), the PCE of the RCZTSSe device exhibited the highest efficiency.

The corresponding EQE curves for RCZTSSe solar cells with various Rb contents are shown in Figure 9d. Compare with the un-doped CZTSSe device, the RCZTSSe device exhibited higher optical response throughout the wavelength region, which could be attributed to the reduction of non-radiative recombination [57]. Additionally, Rb doping can also efficiently suppress the formation of the detrimental Cu_Zn_ defects and related defect clusters, and decrease the route of carrier complexes, thereby enhancing the EQE [58]. A flat and high photoelectric response of nearly 80% was observed for the Rb-2% device within the regions of visible and near-infrared spectra, which indicated that the majority of the deficit induced by the conversion of incident photons into carriers was dealt with, thus enhancing the collection ability of the carriers [59]. It was conducive to the improvement of J_sc_. Based on the EQE results, the RCZTSSe thin-film solar cells exhibited enhanced carrier collection abilities compared to those of the un-doped solar cells.

To verify the enhancement of photovoltaic performance, the statistical distributions of other performance characteristics for the Rb-2% and Rb-0% CZTSSe devices were collected and are shown inFigure 10. It was found that the mean values of V_oc_, J_sc_, and FF of the Rb-2% devices were much larger than those of the Rb-0% devices. The increase in V_oc_ could be ascribed to the inhibition of various copper-related defects in the RCZTSSe films, thereby mitigating the detrimental effects of band tail states. The enhancement of J_sc_ was a result of improved light absorption capabilities. The increase in FF primarily stemmed from the advantageous influence of optimal Rb-doping content on the surface quality of the RCZTSSe films. The outcomes showed that Rb-doped devices significantly outperformed un-doped devices.

## 3. Experimental Methods

### 3.1. Preparation of RCZTSSe Thin Films

**Figure 11:**
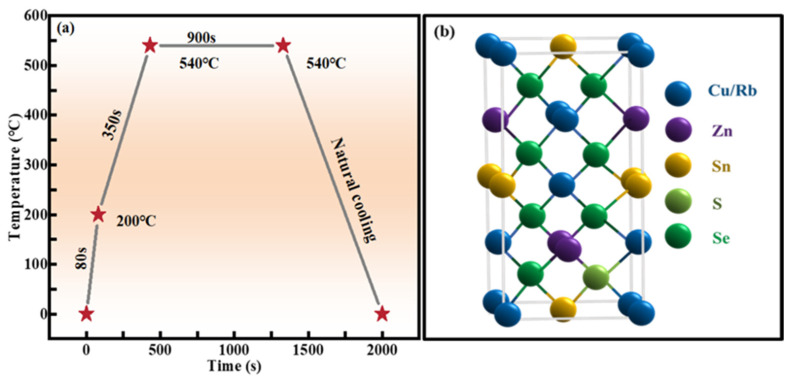
(**a**) Temperature profiles of a two-step selenization process. (**b**) The crystal structure cell of kesterite RCZTSSe.

Firstly, Rb-doped Cu_2_ZnSnS_4_ (RCZTS) thin films were synthesized using the sol–gel technique. Appropriate amounts of C_4_H_6_CuO_4_·H_2_O, ZnCl_2_, SnCl_2_·2H_2_O, CH_4_N_2_S, and RbCl were added to DMSO solvent, and C_2_H_7_NO was added as a stabilizer. The solution underwent magnetic stirring for a duration of 4 h until the chemicals were all dissolved and the liquid was transparent and clarified. The Rb-doping content in the precursor solution was adjusted by altering the proportion of Rb/(Cu + Rb). The mole ratios of Rb/(Cu + Rb) were set to 0%, 1%, 2%, 3%, and 5%, and the precursor solutions with different Rb-doping contents were obtained. Then, Rb-doped CZTS (RCZTS) precursor films were prepared on 2 × 2 cm^2^ soda lime glass (SLG) by the spin coating technique. Next, 0.2 g selenium particles and RCZTS film samples were placed in a chamber made of graphite, heated rapidly to 540 °C in two steps in a N_2_ atmosphere, annealed at this temperature for 15 min, and finally cooled to room temperature to obtain the RCZTSSe films, as shown in Figure 11a. The absorption layer was approximately 1.0 μm thick. The crystal structure of the RCZTSSe thin film is depicted in Figure 11b.

### 3.2. Solar Cell Fabrication

**Figure 12:**
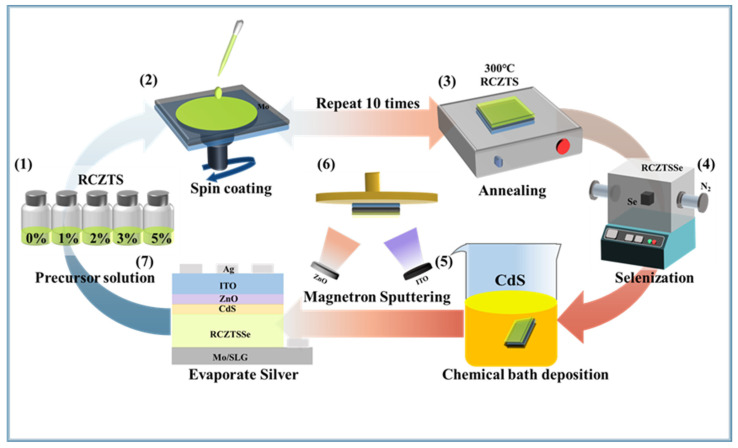
Schematic drawing of the preparation process of an RCZTSSe solar cell. (**1**) Preparing an RCZTS precursor solution with different Rb-doping content; (**2**) spin-coating the precursor solution; (**3**) annealing the RCZTS thin film; (**4**) selenization in a N_2_ atmosphere; (**5**) preparing a CdS layer by chemical bath deposition; (**6**) preparing ZnO and ITO layers by magnetron sputtering; (**7**) preparing a silver electrode.

The RCZTSSe device was prepared using an SLG/Mo/R CZTSSe/CdS/i-ZnO/ITO/Ag structure. As shown in Figure 12, firstly, we introduced a RbCl solution as a Rb source into the precursor solution. The filtered liquid was spin-coated onto Mo-coated SLG operating at 3000 rotations per minute for 30 s, subsequently baked at a temperature of 300 °C on a heated plate to prepare the precursor films. Subsequently, selenium annealing was carried out in a N_2_ atmosphere. A 60 nm-thick CdS buffer layer was deposited at 65 °C via the standard chemical bath deposition (CBD) process, then, a 60 nm i-ZnO layer and a 250 nm ITO layer were fabricated using magnetron sputtering. Finally, a 200 nm-thick silver electrode was prepared using the thermal evaporation technique. The RCZTSSe device was mechanically divided, and the active area of the measuring device was 0.19 cm^2^.

### 3.3. Material and Device Characterization

An X-ray diffractometer (XRD) of Rigaku Company (Rigaku, Tokyo, Japan) was used to explore the crystalline structure of the RCZTSSe thin films. Raman spectra were tracked using a Renishaw spectrometer (Renishaw, London, UK) operating at a specific excitation wavelength of 514 nm. We utilized field emission scanning electron microscopy (FE-SEM, JEOL, Tokyo, Japan) measurements to examine the surface structure of the CZTSSe films. Chemical valence states of Cu, Zn, Sn, S, Se, and Rb were ascertained utilizing X-ray photoelectron spectroscopy (XPS, VG Scienta, Eastgreenstead, East Sussex, UK). The optical bandgap of the samples was evaluated using a UV–Vis spectrophotometer (Shimadzu, Kyoto, Japan). Their electrical characteristics were evaluated using Hall measuring equipment. In this study, we used the Lakeshore 7600 Hall Effect Test System (Lakeshore, Columbus, OH, USA), manufactured in the United States, for testing. The Hall mobility, carrier concentration, and resistivity of the RCZTSSe samples were measured using the four-probe Hall effect measurement method. The current–voltage (J–V) data of the solar cells were fetched by simulating daylight with AM 1.5G illumination. External quantum efficiency (EQE) was obtained by measuring the solar cells with a Zolix Solar Cell Scan100 instrument (Zolix, Beijing, China).

## 4. Conclusions

In conclusion, Rb cations were introduced by adding a RbCl solution into a CZTS precursor solution. Rb cation-doped CZTSSe thin films were synthesized using the sol–gel method accompanied by the post-selenization technique for the first time. A systematic investigation showed that an appropriate amount of Rb doping greatly improved the crystallinity of CZTSSe thin films, enhanced the surface morphology, and accelerated the growth of grains. When the content of Rb was 2%, the average size of the grain of the film was maximal. As the doping content of Rb increased, the E_g_ of RCZSSe thin films gradually increased. Hall measurement showed that the Rb-2% RCZTSSe film had the best electrical performance. Our comprehensive research indicates that Rb doping can significantly decrease the defect density of Cu_Zn_ and [2Cu_Zn_ + Sn_Zn_] defects, thereby improving the light absorption and enhancing charge transport. Finally, on account of the enhanced absorber quality, kinetics of carrier transport, and defect characteristics, Rb-2% was capable of substantial improvements in V_oc_ and J_sc_ concurrently, resulting in an efficiency boost from 5.02% to 7.32%. Overall, this work demonstrates the optimistic function of Rb doping in inhibiting the various copper-related defects and provides more hopeful possibilities for the progress of alkali metal-doped CZTSSe photovoltaic devices.

## Figures and Tables

**Figure 10:**
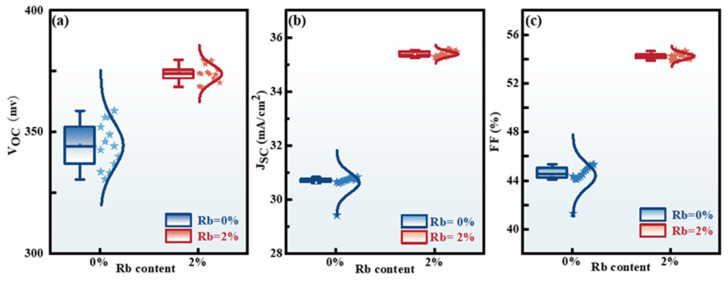
Statistical box diagrams of performance parameters of Rb-0% and Rb-2% devices: (**a**) V_oc_, (**b**) J_sc_, and (**c**) FF.

**Table 1:** Overview of the EDS findings of RCZTSSe thin films with different Rb-doping contents.

x	Composition(%at)						Cu + Rb/(Zn + Sn)	Rb/(Rb + Cu)
	Cu	Zn	Sn	S	Se	Rb		
0	23.96 ± 0.1	17.15 ± 0.28	11.27 ± 0.13	3.15 ± 0.39	44.47 ± 0.91	0	0.84 ± 0.01	0
0.01	23.40 ± 0.03	17.71 ± 0.59	10.15 ± 0.47	2.15 ± 0.49	45.89 ± 0.44	0.70 ± 0.13	0.87 ± 0.01	0.03
0.02	23.26 ± 0.23	17.43 ± 0.45	10.37 ± 0.45	1.99 ± 0.52	45.77 ± 0.63	1.18 ± 0.35	0.88	0.05 ± 0.01
0.03	23.48 ± 0.16	17.27 ± 0.11	9.96 ± 0.29	2.71 ± 0.51	45.23 ± 0.38	1.35 ± 0.12	0.91 ± 0.02	0.05
0.05	22.80 ± 0.45	16.2 ± 0.71	13.18 ± 0.77	2.35 ± 0.01	43.92 ± 0.57	1.55 ± 0.18	0.83 ± 0.02	0.07 ± 0.01

**Table 2:** Electrical properties of the RCZTSSe thin films.

Samples	Resistivity (Ω·cm^2^)	Carrier Concentration (cm^−3^)	Mobility (cm^2^V^−1^s^−1^)	Type
Rb = 0%	9.96	6.26 × 10^17^	2.23 × 10^1^	p
Rb = 1%	2.94 × 10^1^	2.13 × 10^17^	4.83 × 10^1^	p
Rb = 2%	4.91 × 10^1^	1.27 × 10^17^	6.64 × 10^1^	p
Rb = 3%	1.43 × 10^1^	4.37 × 10^17^	1.60 × 10^1^	P
Rb = 5%	1.22	7.28 × 10^18^	7.03 × 10^−1^	P

**Table 3:** The features of RCZTSSe thin-film solar cells with various Rb-doping contents. Overview of device parameters of the RCZTSSe solar cells with various Rb-doping contents.

Device.	Active Area (cm^2^)	V_OC_ (mV)	J_SC_ (mA/cm^−2^)	FF (%)	PCE (%)	R_s_ (Ω·cm^2^)	R_sh_(Ω·cm^2^)
CZTSSe (Rb = 0%)	0.19	358.75 ± 7.37	30.84 ± 0.81	45.33 ± 2.17	5.02 ± 0.44	19.36 ± 1.16	279.76 ± 28.19
CZTSSe (Rb = 1%)	0.19	374.73 ± 12.03	32.33 ± 2.35	50.92 ± 0.72	6.17 ± 0.34	16.84 ± 1.84	641.44 ± 66.31
CZTSSe (Rb = 2%)	0.19	379.69 ± 3.99	35.27 ± 0.16	54.66 ± 0.31	7.32 ± 0.10	12.66 ± 0.47	690.58 ± 21.85
CZTSSe (Rb = 3%)	0.19	371.03 ± 12.72	33.52 ± 3.07	53.12 ± 1.02	6.61 ± 0.65	12.87 ± 2.14	526.30 ± 44.5
CZTSSe (Rb = 5%)	0.19	366.11 ± 3.17	32.32 ± 0.59	45.55 ± 0.13	5.39 ± 0.13	19.78 ± 0.52	272.59 ± 4.92

## Data Availability

The authors do not have permission to share data.
